# *Vital Signs:* Characteristics of Drug Overdose Deaths Involving Opioids and Stimulants — 24 States and the District of Columbia, January–June 2019

**DOI:** 10.15585/mmwr.mm6935a1

**Published:** 2020-09-04

**Authors:** Julie O’Donnell, R. Matt Gladden, Christine L. Mattson, Calli T. Hunter, Nicole L. Davis

**Affiliations:** 1 Division of Overdose Prevention, National Center for Injury Prevention and Control, CDC.

## Abstract

**Introduction:**

Provisional estimates indicate that drug overdose deaths increased in 2019 after a slight decrease in 2018. In 2018, overdose deaths primarily involved opioids, with continued increases in deaths involving illicitly manufactured fentanyls (IMFs). Deaths involving stimulants such as cocaine and methamphetamine are also increasing, mainly in combination with opioids.

**Methods:**

CDC analyzed data on drug overdose deaths during January–June 2019 from 24 states and the District of Columbia (DC) in the State Unintentional Drug Overdose Reporting System to describe characteristics and circumstances of opioid- and stimulant-involved overdose deaths.

**Results:**

Among 16,236 drug overdose deaths in 24 states and DC, 7,936 (48.9%) involved opioids without stimulants, 5,301 (32.6%) involved opioids and stimulants, 2,056 (12.7%) involved stimulants without opioids, and 943 (5.8%) involved neither opioids nor stimulants. Approximately 80% of overdose deaths involved one or more opioid, and IMFs were involved in three of four opioid-involved overdose deaths. IMFs, heroin, cocaine, or methamphetamine (alone or in combination) were involved in 83.8% of overdose deaths. More than three in five (62.7%) overdose deaths had documentation of at least one potential opportunity for overdose prevention intervention.

**Conclusions and implications for public health practice:**

Identifying opportunities to intervene before an overdose death and implementing evidence-based prevention policies, programs, and practices could save lives. Strategies should address characteristics of overdoses involving IMFs, such as rapid overdose progression, as well as opioid and stimulant co-involvement. These efforts should be complemented by efforts to prevent initiation of prescription opioid and stimulant misuse and illicit drug use.

## Introduction

Provisional estimates indicate that drug overdose deaths (overdose deaths) increased in 2019 after a slight decrease from 2017 to 2018 (*1*,*2*).* Approximately two thirds of overdose deaths in 2018 involved an opioid, but the opioid types and combinations contributing to deaths are changing (*1*–*3*). For example, although overdose deaths involving prescription opioids and heroin decreased from 2017 to 2018, those involving synthetic opioids excluding methadone (primarily illicitly manufactured fentanyl [IMF]) and co-involving stimulants increased (*2*,*3*). Deaths co-involving cocaine and IMF, and involving psychostimulants with abuse potential (e.g., methamphetamine) with and without opioids have driven recent increases in stimulant-involved overdose deaths (*3*,*4*). The specific drugs and drug combinations involved in overdose deaths have implications for substance use disorder treatment regimens and outcomes, overdose prevention strategies (e.g., avoidance of using drugs when alone) (*5*), and overdose response (e.g., stimulant use can affect the response to administered naloxone) (*6*).

Targeting common fatal overdose circumstances with effective and promising public health interventions can prevent deaths (*7*). Examples include treating underlying substance use disorder (*8*), targeting important touchpoints to facilitate linkage to treatment (e.g., during treatment for a nonfatal drug overdose or upon release from incarceration) (*9*,*10*), providing mental health treatment (*11*), and expanding community naloxone distribution (*12*).

This report describes decedent demographic characteristics and circumstances surrounding overdose deaths during January–June 2019 among 25 jurisdictions participating in CDC’s State Unintentional Drug Overdose Reporting System (SUDORS),^†^ and it highlights the involvement of opioids and stimulants, separately and in combination.

## Methods

Twenty-one jurisdictions participating in SUDORS reported all unintentional and undetermined intent overdose deaths that occurred during January–June 2019; four additional states reported overdose deaths in a subset of counties.^§^^,^^¶^ Jurisdictions abstract data from death certificates and medical examiner/coroner reports, including death scene investigation findings and all drugs detected by postmortem toxicology testing. Detected drugs were classified as involved in (i.e., contributing to) overdose deaths if the medical examiner/coroner listed them as causing death on the death certificate or in the medical examiner/coroner report.**

Overdose deaths were grouped by opioid and stimulant involvement into four mutually exclusive categories: 1) opioids without stimulants, 2) opioids and stimulants, 3) stimulants without opioids, and 4) neither opioids nor stimulants. Also, overdose deaths were grouped into the 10 most frequently occurring mutually exclusive combinations of opioid type or types (illicitly manufactured fentanyls^††^ [referred to as IMFs, which include fentanyl and fentanyl analogs], heroin,^§§^ prescription opioids,^¶¶^ other illicit synthetic opioids [e.g., U-47700]), and stimulant type or types (cocaine, methamphetamine, other illicit stimulants [e.g., MDMA], and prescription stimulants***). Overdose death combinations included deaths involving one drug type (e.g., involving IMFs without other opioid or stimulant involvement) and deaths involving two or more types (e.g., co-involved IMFs and cocaine), but did not reflect nonopioid, nonstimulant drug involvement (e.g., benzodiazepines). The following potential intervention opportunities (per evidence^†††^ in the medical examiner/coroner report) were assessed: 1) recent institutional release (<1 month),^§§§^ 2) previous nonfatal overdose, 3) mental health diagnosis, 4) ever having been treated for substance use disorder, 5) bystander present when fatal overdose occurred, and 6) fatal drug use witnessed.

Frequencies and percentages of decedent demographics, overdose location,^¶¶¶^ geographic region**** of the jurisdictions, and potential opportunities for intervention were stratified by opioid/stimulant involvement. Pairwise chi-squared testing was used to detect statistically significant differences (p<0.01) among percentages. Because of the potential for incomplete data, the analysis of potential opportunities for intervention only included deaths with overdose-specific circumstances noted in the medical examiner/coroner report (15,295; 94.2% of overdose deaths). Analyses were conducted using SAS statistical software (version 9.4; SAS Institute).

## Results

**FIGURE 1:**
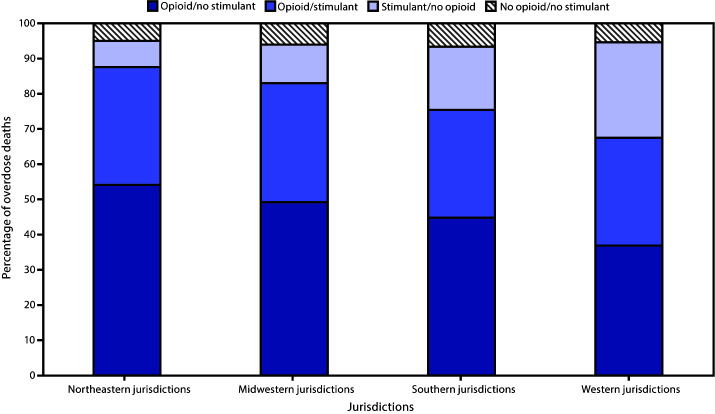
Distribution of opioid/stimulant involvement in drug overdose deaths, by geographic region* — State Unintentional Drug Overdose Reporting System (SUDORS), 25 jurisdictions, January–June 2019^†^ *Midwestern: Illinois, Indiana, Michigan, Minnesota, Ohio, and Wisconsin; *Northeastern*: Connecticut, Maine, Massachusetts, New Jersey, Pennsylvania, Rhode Island, and Vermont; *Southern*: Delaware, District of Columbia, Georgia, Kentucky, North Carolina, Oklahoma, Tennessee, and West Virginia; *Western*: Alaska, Nevada, Utah, and Washington. ^†^ Pairwise chi-squared testing found statistically significant differences (p<0.01) for each pairwise comparison of regions.

Twenty-five jurisdictions reported 16,236 overdose deaths during January–June 2019. Among these, 7,936 (48.9%) involved opioids without stimulants, 5,301 (32.6%) involved opioids and stimulants, 2,056 (12.7%) involved stimulants without opioids, and 943 (5.8%) involved neither opioids nor stimulants (Table). In all regions, overdose deaths involving opioids without stimulants were most common (36.9%–54.1%), followed by deaths involving opioids and stimulants (30.6%–33.8%), then deaths involving stimulants without opioids (7.4%–27.1%) (Figure 1). This pattern was most prominent in Northeastern and Midwestern jurisdictions, where deaths involving opioids (with or without stimulants) accounted for 87.6% and 83.0%, respectively, of all overdose deaths.

**TABLE:** Demographic characteristics of decedents, location of overdose, and drug type involved in drug overdose deaths, by opioid/stimulant involvement — State Unintentional Drug Overdose Reporting System (SUDORS), 25 jurisdictions, January–June 2019

Characteristic	No. (%)				
	All drug overdose deaths	Categories of opioid/stimulant involvement			
		Opioids/No stimulants	Opioids/Stimulants	Stimulants/No opioids	No opioids or stimulants
**No. (%) of all overdose deaths**	16,236 (100)	7,936 (48.9)	5,301 (32.6)	2,056 (12.7)	943 (5.8)
Sex*^,†^					
Male	11,117 (68.5)	5,487 (69.1)	3,652 (68.9)	1,482 (72.1)	496 (52.6)
Female	5,118 (31.5)	2,448 (30.9)	1,649 (31.1)	574 (27.9)	447 (47.4)
Race/Ethnicity*^,§^					
White, non-Hispanic	12,104 (75.2)	6,180 (78.5)	3,825 (72.7)	1,318 (65.2)	781 (83.3)
Black, non-Hispanic	2,553 (15.9)	1,002 (12.7)	945 (18.0)	507 (25.1)	99 (10.6)
Other, non-Hispanic	359 (2.2)	144 (1.8)	118 (2.2)	79 (3.9)	18 (1.9)
Hispanic	1,076 (6.7)	545 (6.9)	373 (7.1)	118 (5.8)	40 (4.3)
Age group, yrs*^,§^					
<15	19 (0.1)	—^¶^	—^¶^	—^¶^	—^¶^
15–24	930 (5.7)	530 (6.7)	293 (5.5)	63 (3.1)	44 (4.7)
25–34	4,017 (24.7)	2,079 (26.2)	1,491 (28.1)	286 (13.9)	161 (17.1)
35–44	4,112 (25.3)	1,960 (24.7)	1,529 (28.8)	421 (20.5)	202 (21.4)
45–54	3,585 (22.1)	1,656 (20.9)	1,136 (21.4)	579 (28.2)	214 (22.7)
55–64	2,871 (17.7)	1,364 (17.2)	733 (13.8)	566 (27.5)	209 (22.2)
≥65	701 (4.3)	336 (4.3)	115 (2.2)	141 (6.9)	109 (11.6)
Location of overdose*^,§^					
Any home setting	12,705 (82.4)	6,484 (85.0)	4,052 (79.9)	1,506 (78.3)	663 (84.2)
Decedent's own home	9,779 (63.5)	5,198 (68.1)	2,893 (57.1)	1,156 (60.1)	532 (67.6)
Home setting but not decedent's home	2,926 (19.0)	1,286 (16.9)	1,159 (22.9)	350 (18.2)	131 (16.6)
Any nonhome setting	2,705 (17.6)	1,145 (15.0)	1,018 (20.1)	418 (21.7)	124 (15.8)
Hotel/Motel	711 (4.6)	265 (3.5)	344 (6.8)	77 (4.0)	25 (3.2)
Motor vehicle	423 (2.7)	186 (2.4)	160 (3.2)	66 (3.4)	12 (1.5)
Supervised residential facility	220 (1.4)	145 (1.9)	53 (1.0)	12 (0.6)	11 (1.4)
Other	1,351 (8.8)	549 (7.2)	461 (9.1)	263 (13.7)	78 (9.9)
Evidence of route of drug use*^,^**					
Injection^§^	4,212 (27.3)	2,138 (28.1)	1,782 (34.8)	246 (12.6)	46 (6.3)
Smoking^††^	1,415 (9.2)	385 (5.1)	753 (14.7)	255 (13.0)	22 (3.0)
Ingestion^§§^	2,267 (14.7)	1,265 (16.6)	616 (12.0)	208 (10.6)	178 (24.3)
Snorting/Sniffing^†^	1,651 (10.7)	875 (11.5)	639 (12.5)	120 (6.1)	17 (2.3)
Other route	107 (0.7)	—^¶^	—^¶^	—^¶^	—^¶^
No information about route^¶¶^	7,724 (50.1)	3,707 (48.7)	2,222 (43.4)	1,298 (66.4)	498 (67.9)
Opioid involvement***					
Any opioids	13,237 (81.5)	7,936 (100.0)	5,301 (100.0)	N/A	N/A
IMFs^§^	9,988 (61.5)	5,727 (72.2)	4,261 (80.4)	N/A	N/A
Heroin^§^	4,579 (28.2)	2,606 (32.8)	1,973 (37.2)	N/A	N/A
Prescription opioids^§^	3,354 (20.7)	2,440 (30.7)	914 (17.2)	N/A	N/A
Other illicit synthetic opioids^§^	12 (0.1)	—^¶^	—^¶^	N/A	N/A
Stimulant involvement***					
Any stimulants	7,357 (45.3)	N/A	5,301 (100.0)	2,056 (100.0)	N/A
Cocaine^§^	4,598 (28.3)	N/A	3,633 (68.5)	965 (46.9)	N/A
Methamphetamine^§^	2,857 (17.6)	N/A	1,766 (33.3)	1,091 (53.1)	N/A
Prescription stimulants^§^	329 (2.0)	N/A	272 (5.1)	57 (2.8)	N/A
Other illicit stimulants^†††^	69 (0.4)	N/A	46 (0.9)	23 (1.1)	N/A
**Involvement of common illicit drugs (IMFs, heroin, cocaine, and methamphetamine)**					
IMFs or heroin^§^	11,197 (69.0)	6,351 (80.0)	4,846 (91.4)	N/A	N/A
Cocaine or methamphetamine^§^	7,106 (43.8)	N/A	5,101 (96.2)	2,005 (97.5)	N/A
IMFs, heroin, cocaine, or methamphetamine^§^	13,605 (83.8)	6,351 (80.0)	5,249 (99.0)	2,005 (97.5)	N/A
1 of these 4 drugs involved^§^	6,824 (50.2)	4,369 (68.8)	501 (9.5)	1,954 (97.5)	N/A
2 or more of the 4 drugs involved^§^	6,781 (49.8)	1,982 (31.2)	4,748 (90.5)	51 (2.5)	N/A

**Abbreviations:** IMFs = illicitly manufactured fentanyls; N/A = not applicable.

* Numbers might not sum to the overall totals because of missing values excluded (sex: 1 missing value; race/ethnicity: 144 missing values; age group: 1 missing value; location of overdose: 826 missing values); percentages might not sum to 100% because of rounding or because routes of drug use are not mutually exclusive.

^†^ Pairwise chi-squared testing found statistically significant differences (p<0.01) for all comparisons except opioid/no stimulant versus opioid/stimulant.

^§^ Pairwise chi-squared testing found statistically significant differences (p<0.01) for all comparisons.

^¶^ Data suppressed because cell contained fewer than 10 deaths or to prevent calculation of another suppressed cell.

** Sample limited to deaths for which the medical examiner/coroner report was available and at least one overdose-specific circumstance field was abstracted. N = 15,415 (94.9% of the total 16,236 sample).

^††^ Pairwise chi-squared testing found statistically significant differences (p<0.01) for all comparisons except opioid/no stimulant versus no opioid/no stimulant and stimulant/opioid versus stimulant/no opioid.

^§§^ Pairwise chi-squared testing found statistically significant differences (p<0.01) for all comparisons except stimulant/opioid versus stimulant/no opioid.

^¶¶^ Pairwise chi-squared testing found statistically significant differences (p<0.01) for all comparisons except stimulant/no opioid versus no opioid/no stimulant.

*** Specific opioids and stimulants listed are not mutually exclusive, so percentages will not sum to 100%. Of the deaths involving any opioid, 171 were not classified into one of the listed opioid types because of lack of specificity.

^†††^ Pairwise chi-squared testing found no statistically significant differences (p<0.01) for stimulant/opioid versus stimulant/no opioid comparison.

More than two thirds (68.5%) of decedents were male, and three quarters (75.2%) were non-Hispanic White (Table). Among overdose deaths involving opioids (with and without stimulants), most decedents (53.3%) were aged 25–44 years; among overdose deaths involving stimulants without opioids, most decedents (55.7%) were aged 45–64 years. Evidence of injection drug use^††††^ was more common among opioid-involved deaths than among deaths that did not involve opioids.

Most overdose deaths (83.8%) involved one or more of four illicit drugs (IMFs [61.5%], cocaine [28.3%], heroin [28.2%], or methamphetamine [17.6%]) (Table); nearly one half (49.8%) of these deaths involved two or more of those drugs. IMFs were involved in 80.4% of opioid overdose deaths with stimulants and in 72.2% without stimulants. Heroin was involved in 34.6% of opioid overdose deaths, and 73.6% of heroin overdose deaths co-involved IMFs (data not shown). Either cocaine or methamphetamine was involved in nearly all stimulant overdose deaths (96.2% with opioids, 97.5% without). Prescription opioids were involved more often in deaths involving opioids without stimulants (30.7%) than in those with stimulants (17.2%).

**FIGURE 2:**
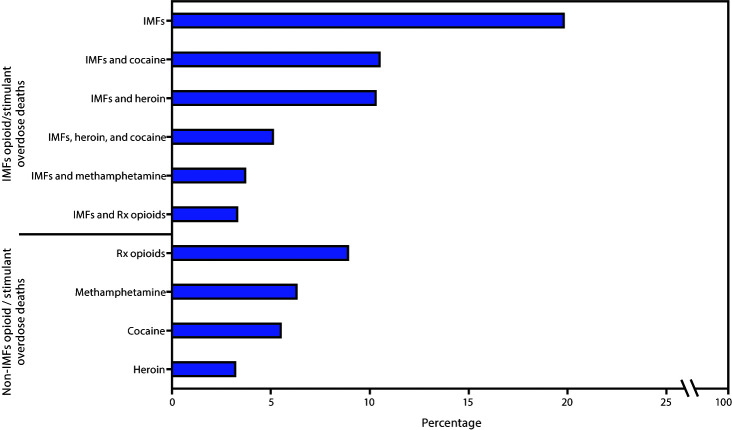
Percentage of drug overdose deaths involving the 10 most common combinations of opioids and stimulants (mutually exclusive), by involvement of illicitly manufactured fentanyls (IMFs) — State Unintentional Drug Overdose Reporting System (SUDORS), 25 jurisdictions, January–June 2019*^,^^†^ **Abbreviation:** Rx = prescription. * Drug overdose deaths involving IMFs with no other opioids or stimulants was the most frequent combination among Northeastern (24.3%), Midwestern (21.2%), and Southern (15.4%) jurisdictions. ^†^ Drug overdose deaths involving methamphetamine with no other opioids or stimulants was the most frequent combination among Western jurisdictions (22.1%).

The 10 most frequently occurring opioid and stimulant combinations accounted for 76.9% of overdose deaths (Figure 2). Six drug combinations, including the three most common, involved IMFs and 1) no other opioid or stimulant (19.8% of deaths), 2) cocaine (10.5%), 3) heroin (10.3%), 4) heroin and cocaine (5.1%), 5) methamphetamine (3.7%), and 6) prescription opioids (3.3%). Deaths without IMFs involved a single opioid without other opioids or stimulants (only prescription opioids [9.2%], only heroin [3.2%]) or a single stimulant without other opioids or stimulants (only methamphetamine [6.3%], only cocaine [5.5%]).

**FIGURE 3:**
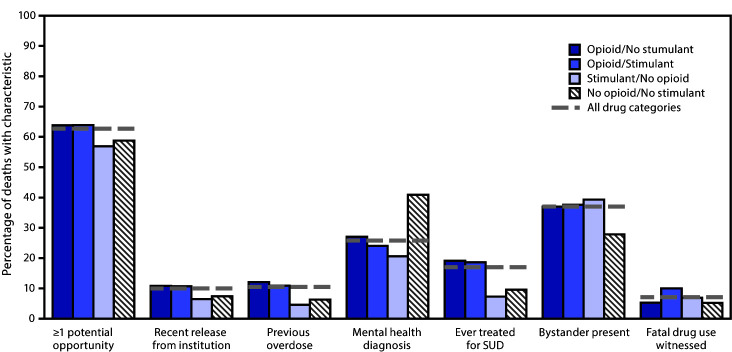
Potential opportunities for intervention, by opioid/stimulant involvement — State Unintentional Drug Overdose Reporting System (SUDORS), 25 jurisdictions, January–June 2019*^,^^†^^,^^§^^,^^¶^^,^** **Abbreviation:** SUD = substance use disorder. * Sample for this figure limited to deaths for which the medical examiner/coroner report was available, at least one overdose-specific circumstance field was abstracted, and none of the fields for characteristics had missing data. N = 15,295 (94.2% of the total 16,236 sample). **^†^** Pairwise chi-squared testing for at least one potential opportunity, recent release from institution, previous overdose, and ever treated for SUD found statistically significant differences (p<0.01) for all comparisons except opioid/no stimulant versus opioid/stimulant and stimulant/no opioid versus no opioid/no stimulant. ^§^ Pairwise chi-squared testing for mental health diagnosis found statistically significant differences (p<0.01) for all comparisons. ^¶^ Pairwise chi-squared testing for bystander present found statistically significant differences (p<0.01) for opioid/no stimulant, opioid/stimulant, and stimulant/no opioid versus no opioid/no stimulant. ** Pairwise chi-squared testing for fatal drug use witnessed found statistically significant differences (p<0.01) for all comparisons except opioid/no stimulant versus no opioid/no stimulant and stimulant/no opioid versus no opioid/no stimulant.

More than three in five overdose deaths (62.7%) had evidence of at least one potential opportunity for intervention (Figure 3). Approximately one in ten opioid overdose deaths had evidence of past-month institutional release (10.7% with stimulants; 10.8% without stimulants) or previous overdose (10.9%; 12.1%). Mental health diagnoses were documented for one quarter (25.8%) of overdose deaths. Evidence of current or past substance use disorder treatment was more common among opioid overdose deaths (18.6% with stimulants; 19.1% without stimulants) than nonopioid overdose deaths (<10%). Among overdose deaths, 37% occurred with a bystander present.

## Discussion

This report provides three critical insights that can inform overdose prevention efforts. First, approximately 80% of overdose deaths involved opioids, and three of four opioid overdose deaths involved IMFs. The supply of IMFs and overdose deaths involving synthetic opioids excluding methadone (primarily IMFs) are projected to have increased for the seventh straight year in 2019 (*1*).^§§§§^ Second, IMFs, heroin, cocaine, or methamphetamine (alone or in combination) were involved in nearly 85% of overdose deaths. Complicating intervention and treatment efforts, one half of these deaths involved two or more of these four drugs. Third, potential opportunities for intervention, which could be targeted for overdose prevention, were documented in approximately 60% of overdose deaths.

Interventions should address characteristics of overdoses involving IMFs. First, IMFs can be highly potent (e.g., fentanyl has 50–100 times the potency of morphine; carfentanil has 30–100 times the potency of fentanyl) (*13*), and use might quickly progress to overdose (*5*,*14*), especially when injected. Consequently, improving overdose response time by expanding community naloxone distribution, increasing naloxone prescribing and dispensing from pharmacies, and encouraging persons to not use drugs when alone might reduce IMF overdose deaths (*5*,*12*). Second, powdered IMFs are often sold as or mixed with white powdered heroin (primarily east of the Mississippi River) with or without the knowledge of the person buying the products, but deaths involving IMFs and products containing IMFs are less prevalent in western black tar heroin markets.^¶¶¶¶^ Mixing of IMFs into heroin, and in some places IMFs supplanting the heroin supply, is increasing over time, consistent with findings that more than seven in 10 (73.6%) heroin-involved overdose deaths co-involved IMFs. Pressing IMFs into counterfeit prescription pills resembling both prescription opioids and other drugs (e.g., benzodiazepines) has allowed IMFs to spread into additional drug markets. IMFs are difficult to mix consistently, resulting in possibly varying concentrations of IMFs between and within products, or persons might use IMFs when expecting to use heroin, other opioids, or (rarely) nonopioids; either could increase the risk for overdose.***** Interventions conducted by risk reduction organizations (e.g., syringe services programs) to reduce overdoses among persons exposed to IMFs (e.g., naloxone distribution) and to link populations at high risk (e.g., persons who inject drugs) with prevention and treatment services might mitigate these overdose risks (*15*).^†††††^ Finally, timely response by public health and public safety officials to growing threats such as mixing of IMFs in nonopioid products, and outbreaks involving fentanyl analogs (e.g., carfentanil) is warranted.^§§§§§^

In this report, one third (32.6%) of overdose deaths co-involved opioids and stimulants. Co-use of opioids and stimulants elevates fatal overdose risk and is associated with poorer medical, mental health, and substance use disorder treatment outcomes (*16*). Supporting increased access to medications for opioid use disorder^¶¶¶¶¶^ and evidence-based treatments for stimulant use disorders (*17*) can help mitigate risks. Research into more effective treatments for co-occurring opioid and stimulant use disorder is also needed. Methamphetamine was involved in approximately one half of stimulant overdose deaths without opioids. The methamphetamine supply has increased substantially since 2011,****** with accompanying increases in methamphetamine-related treatment admissions (*18*) and overdose deaths involving psychostimulants with abuse potential (e.g., methamphetamine) (*1*,*4*). Tracking of and response to these increases might help prevent further deaths.

Public health interventions targeting overdose risk factors identified in this report have shown effectiveness, especially for opioid overdose prevention (*7*). Recent release from an institution and previous overdose were both reported for approximately one in 10 opioid overdose deaths. Initiating or continuing medications for opioid use disorder among persons leaving prison (*7*,*10*) and expanding linkage to care programs targeting persons treated for a nonfatal overdose (*7*,*9*) can mitigate overdose risk. Also, outreach to groups at higher risk for overdose (e.g., persons who inject drugs) shows promise in reducing drug overdose deaths (*7*,*15*). For one quarter of deaths, there was evidence of a mental health diagnosis. Integrating substance use disorder and mental health treatment can improve treatment outcomes, which could help reduce drug overdoses (*11*,*19*). Finally, presence of a bystander at nearly four in 10 opioid- and stimulant-involved overdose deaths suggests a need to increase bystander naloxone training, access, and use (*5*,*12*). CDC, through the Overdose Data to Action program, is supporting expansions of programs linking persons at risk for overdose to treatment and risk reduction programs.

The findings in this report are subject to at least five limitations. First, the 25 jurisdictions are not nationally representative, and four states reported a subset of overdose deaths. Western states are underrepresented, likely resulting in an underestimation of methamphetamine overdose deaths that more frequently occur in the West (*20*). Second, toxicology testing and drug involvement determination varies over time and across jurisdictions. Third, all drugs detected are listed as involved when the cause of death does not specify drugs (e.g., multitoxicity death), which might overestimate drug involvement. Testing, drug involvement determination, and coding biases are minimized by focusing on commonly tested drugs frequently involved in deaths. Fourth, medical examiner/coroner reports likely underestimate intervention opportunities as investigators might have limited information. Finally, details about potential opportunities for intervention were limited (e.g., no information about whether a decedent was referred to treatment after a prior overdose), and they should therefore not necessarily be interpreted as missed opportunities.

Drug overdose interventions should address the combination and lethality of drugs being used (e.g., IMFs in combination with stimulants) and also work to prevent initiation of prescription drug misuse (e.g., inappropriate prescribing) and illicit drug use. The finding of this report that nearly 85% of overdose deaths involved IMFs, heroin, cocaine, or methamphetamine reflects rapid and continuing increases in the supply of IMFs and methamphetamine, coupled with illicit co-use of opioids and stimulants. This report also highlights important intervention opportunities for persons who use illicit drugs (especially IMFs), including the presence of bystanders, recent release from institutions, and high-risk routes of drug use (e.g., injection) that can be targeted to both prevent overdoses (e.g., by enhancing linkage to evidence-based treatment and risk reduction services) and improve response to overdoses to prevent deaths.

**Summary**

**What is already known about this topic?**

After decreasing from 2017 to 2018, provisional data indicate that drug overdose deaths increased in 2019, driven by opioid-involved and stimulant-involved overdose deaths.

**What is added by this report?**

Illicitly manufactured fentanyls (IMFs), heroin, cocaine, or methamphetamine (alone or in combination) were involved in 83.8% of overdose deaths during January–June 2019; at least one potential opportunity for intervention was identified in 62.7% of overdose deaths.

**What are the implications for public health practice?**

Targeting crucial opportunities for intervention with evidence-based overdose prevention programs can help reverse increases in drug overdose deaths. Interventions to reduce overdose deaths involving illicit opioids and stimulants, particularly IMFs, are needed and should be complemented by efforts to prevent initiation of prescription drug misuse and illicit drug use.

## Footnotes

* https://www.cdc.gov/nchs/nvss/vsrr/drug-overdose-data.htm.

† SUDORS began in 2016 as part of CDC’s Enhanced State Opioid Overdose Surveillance (ESOOS) program, which funded 12 states, with an additional 20 states and the District of Columbia (DC) funded in 2017 to abstract data on opioid overdose deaths. In 2019, SUDORS expanded to collect data on all drug overdose deaths from 47 states and DC (collectively referred to as jurisdictions) as part of CDC’s Overdose Data to Action (OD2A) program. https://www.cdc.gov/drugoverdose/od2a/index.html.

§ Alaska, Connecticut, DC, Delaware, Georgia, Kentucky, Maine, Massachusetts, Michigan, Minnesota, Nevada, New Jersey, North Carolina, Ohio, Oklahoma, Rhode Island, Tennessee, Utah, Vermont, West Virginia, and Wisconsin reported data on all overdose deaths within the jurisdiction. Illinois, Indiana, Pennsylvania, and Washington reported data from a subset of counties that accounted for 86.6%–88.7% of all unintentional and undetermined intent drug overdose deaths in those states in 2017 (SUDORS funding requirement was to report data from counties accounting for ≥75% of the drug overdose deaths in the state in 2017, the most recent year of statewide data available at the time of funding).

¶ Data are reported to SUDORS in half-year increments (January–June and July–December) based on when deaths occurred. Jurisdictions that participated in SUDORS under ESOOS (32 states and DC) were eligible to report data for deaths that occurred during January–June 2019. Twenty-five of the 33 jurisdictions eligible to report data for that period submitted complete data at the time of analysis and were included in this report. Data for this report were downloaded on July 7, 2020, and might differ from other reports because death data might be updated over time, and SUDORS supplements death certificate data with medical examiner/coroner reports.

** When the cause of death indicated multiple drugs were involved but did not indicate specific drugs, all drugs detected by postmortem toxicology testing were classified as involved in the drug overdose death. For example, if the cause of death was “multidrug overdose” and toxicology results were positive for five drugs, all five were classified as involved.

†† Fentanyl was classified as likely illicitly manufactured or likely prescription using toxicology, scene, and witness evidence. In the absence of sufficient evidence to classify fentanyl as illicit or prescription (<7% of deaths involving fentanyl), it was classified as illicit because the vast majority of fentanyl overdose deaths involve illicit fentanyl. With few exceptions, fentanyl analogs are considered illicit because they do not have a legitimate medical use in humans. The three fentanyl analogs with legitimate human medical use are alfentanil, remifentanil, and sufentanil. Fewer than 10 deaths involved any of these three analogs, and they were classified as prescription opioids rather than illicit fentanyl. All other fentanyl analogs were included in the category of illicitly manufactured fentanyls.

§§ If morphine was detected along with 6-acetylmorphine (a metabolite of heroin indicating heroin use), it was classified as heroin. Detection of morphine in the absence of 6-acetylmorphine was classified as likely heroin using toxicology evidence of heroin impurities or other illicit drugs detected or scene or witness evidence that indicated injection drug use, illicit drug use, or a history of heroin use.

¶¶ Drugs coded as prescription opioids were alfentanil, buprenorphine, codeine, dextrorphan, hydrocodone, hydromorphone, levorphanol, loperamide, meperidine, methadone, morphine, noscapine, oxycodone, oxymorphone, pentazocine, prescription fentanyl, propoxyphene, remifentanil, sufentanil, tapentadol, and tramadol. Also included as prescription opioids were brand names (e.g., Opana) and metabolites (e.g., nortramadol) of these drugs and combinations of these drugs and nonopioids (e.g., acetaminophen-oxycodone). Morphine was included as prescription only if scene or witness evidence did not indicate likely heroin use, and if 6-acetylmorphine was not also detected.

*** Drugs coded as prescription stimulants were amphetamine (in the absence of methamphetamine), atomoxetine, ephedrine, and methylphenidate.

††† Reported evidence of decedent and overdose characteristics in SUDORS is likely an underestimation of the true prevalence of those characteristics because SUDORS uses information from medical examiner/coroner reports, which are completed for death investigations, not specifically for SUDORS, and therefore might not reflect all information about the deaths or decedents.

§§§ Release within the month before death from institutional settings, such as prisons/jails, residential treatment facilities, and psychiatric hospitals.

¶¶¶ This is the location where the overdose occurred such as decedent’s home, the home of a person other than the decedent, or a motor vehicle.

**** Jurisdictions were grouped as Midwestern (Illinois, Indiana, Michigan, Minnesota, Ohio, Wisconsin), Northeastern (Connecticut, Maine, Massachusetts, New Jersey, Pennsylvania, Rhode Island, Vermont), Southern (DC, Delaware, Georgia, Kentucky, North Carolina, Oklahoma, Tennessee, West Virginia), or Western (Alaska, Nevada, Utah, Washington), according to U.S. Census region groupings. This report includes 50% of jurisdictions in the Midwest region, 78% of those in the Northeastern region, 47% of those in the Southern region, and 31% of those in the Western region, so groupings should not be interpreted as fully representative of the corresponding Census regions.

†††† Route of drug use is likely underestimated, because physical evidence varies among routes (e.g., syringes/needles as evidence of injection and pipes as evidence of smoking) and can be subject to scene-cleaning by bystanders before death investigations. High percentages of deaths with no information about route of drug use result from lack of physical or witness evidence, lack of documentation of evidence, or data entry error.

§§§§ https://www.nflis.deadiversion.usdoj.gov/DesktopModules/ReportDownloads/Reports/13408NFLISDrugMidYear2019.pdf; https://www.cdc.gov/nchs/nvss/vsrr/drug-overdose-data.htm.

¶¶¶¶ https://www.dea.gov/sites/default/files/2020-01/2019-NDTA-final-01-14-2020_Low_Web-DIR-007-20_2019.pdf.

***** https://www.dea.gov/sites/default/files/2020-01/2019-NDTA-final-01-14-2020_Low_Web-DIR-007-20_2019.pdf.

††††† https://www.cdc.gov/ssp/syringe-services-programs-summary.html.

§§§§§ https://www.dea.gov/sites/default/files/2020-01/2019-NDTA-final-01-14-2020_Low_Web-DIR-007-20_2019.pdf; https://emergency.cdc.gov/han/han00413.asp.

¶¶¶¶¶ https://www.hhs.gov/opioids/about-the-epidemic/hhs-response/better-access/index.html.

****** https://www.nflis.deadiversion.usdoj.gov/DesktopModules/ReportDownloads/Reports/13408NFLISDrugMidYear2019.pdf.
